# Anti-Müllerian Hormone Level Determinants among Non-Polycystic-Ovary-Syndrome Women Undergoing In Vitro Fertilization: A Retrospective Cross-Sectional Study

**DOI:** 10.3390/medicina60091387

**Published:** 2024-08-24

**Authors:** Melinda Kolcsar, László Szabó, Renáta Mihály, Erzsébet Rozália Vass, Zsolt Gáll

**Affiliations:** 1 Department of Pharmacology and Clinical Pharmacy, Faculty of Pharmacy, George Emil Palade University of Medicine, Pharmacy, Science and Technology of Targu Mures, Gheorghe Marinescu Street 38, 540142 Targu Mures, Romania; melinda.kolcsar@umfst.ro; 2 Department of Endocrinology, Mures County Hospital, Gheorghe Marinescu Street 42, 540142 Targu Mures, Romania; szabolaszlo96@gmail.com; 3 Department of Endocrinology, Faculty of Medicine, George Emil Palade University of Medicine, Pharmacy, Science and Technology of Targu Mures, Gheorghe Marinescu Street 38, 540142 Targu Mures, Romania; mihalyrenata@yahoo.com; 4 Department of Gynecology, Zygota Fertility Clinic Mures, Strada Transilvaniei 161, 547530 Sângeorgiu de Mureș, Romania; vasserzsebet948@gmail.com

**Keywords:** anti-Müllerian hormone, age, body mass index, thyroid hormones, autoimmune thyroiditis, sexual hormones, 25-hydroxivitamin D

## Abstract

*Background and Objectives*: The anti-Müllerian hormone (AMH) is a crucial biomarker in regulating ovarian follicle development and female fertility. AMH levels predict ovarian responses in in vitro fertilization (IVF) cycles, helping clinicians tailor treatment strategies. This study aims to determine whether thyroid autoimmunity, age, body mass index (BMI), sexual hormone levels, and 25-hydroxyvitamin D levels influence serum AMH in non-polycystic-ovary-syndrome (PCOS) euthyroid women. *Materials and Methods*: This retrospective cross-sectional study examined 52 female patients at Zygota Fertility Clinic between 2018 and 2022. Women aged 20–45 years with regular menstrual cycles were included, while conditions such as abnormal thyroid-stimulating hormone (TSH) levels, PCOS, and systemic autoimmune diseases were excluded. A number of parameters were measured in the study, including the subjects’ age, BMI, 25-hydroxyvitamin D, serum free thyroxine (fT4), TSH, various antibodies, and a range of reproductive hormones. An analysis of the relationships between AMH and other variables was conducted using Spearman’s correlation coefficient, and an assessment of the impact of confounding factors on AMH levels was conducted using a multivariable linear regression model. *Results*: The results revealed significant negative correlations between AMH levels and age (rho: −0.484, *p* < 0.001) and follicle-stimulating hormone (FSH) (rho: −0.550, *p* < 0.001), while positive correlations existed between AMH and estradiol (rho: 0.352, *p* = 0.011) and total testosterone (rho: 0.542, *p* < 0.001). No significant correlations were found between AMH levels and BMI, LH, or 25-hydroxyvitamin D. *Conclusions*: In this study, ovarian reserve was influenced by age, estradiol, and total testosterone in non-PCOS euthyroid women undergoing IVF. Conversely, BMI and vitamin D status did not significantly impact AMH levels. In order to better understand and possibly manage ovarian reserve, a holistic approach is absolutely essential, taking into account age, weight, hormonal balance, nutrition, and thyroid health.

## 1. Introduction

The anti-Müllerian hormone (AMH) is a member of the transforming growth factor beta (TGF-β) family. Its structure comprises a large N-terminal prodomain and a smaller C-terminal mature signaling domain. It inhibits the development of male reproductive structures during fetal development and also serves as a biomarker for ovarian reserve in women [1]. AMH plays a crucial role in reproductive biology, particularly in regulating ovarian follicle development and determining female fertility. AMH levels are valuable for predicting ovarian response to stimulation protocols in in vitro fertilization (IVF) cycles [2,3]. Evaluating AMH levels before initiating IVF treatment can help predict the ovarian response, including poor and hyper-responses, which is essential for optimizing treatment strategies [4,5]. Research has shown that serum AMH levels correlate with the number of follicles grown and oocytes retrieved, making them reliable predictors of poor ovarian response in IVF cycles [6]. Additionally, AMH levels have been associated with early miscarriage risk in IVF cycles, underscoring the importance of assessing AMH levels before treatment [5,7]. Various factors can influence AMH levels and fertility outcomes. Research indicates that there is a negative correlation between age and AMH levels in healthy women and in those experiencing infertility due to various causes [8,9,10,11,12]. An inverse relationship was reported between body mass index (BMI) and AMH levels, especially in patients with insulin-resistance and polycystic ovary syndrome (PCOS) [13,14]. However, there are studies with small sample sizes that did not find a reduction in AMH levels in obese, middle-aged, infertile women [15]. Several studies have described a positive correlation between adequate vitamin D levels and higher levels of AMH, particularly in late-reproductive-aged females or when compared to fertile women [16,17]. Despite the essential role of vitamin D in female reproductive health, a recent meta-analysis and another retrospective study evidenced no direct relationship between 25-hydroxivitamin D (25-OH vitamin D) and AMH levels [18,19]. The findings are controversial and highlight the need for further studies to determine the optimal 25-OH vitamin D levels during the reproductive period and whether supplementation is necessary for reproductive processes. The impact of hormonal factors, including follicle-stimulating hormone (FSH), thyroid-stimulating hormone (TSH), and androgens, is considered to influence AMH levels and ovarian reserve, as suggested by some studies [14,20,21,22]. Additionally, some conditions such as PCOS and thyroid autoimmunity have been associated with potential alterations in AMH levels, indicating a complex relationship between hormonal factors and fertility [23,24]. AMH not only serves as a marker of ovarian reserve but plays a critical role in regulating reproductive processes. Elevated levels of AMH may disrupt the hypothalamic network regulating reproduction, potentially contributing to conditions like PCOS. Furthermore, AMH is implicated in the central regulation of gonadal function, indicating a broader impact beyond ovarian function [25]. AMH is produced by the granulosa cells of ovarian follicles and is used to assess the number of primordial follicles in the ovaries, which reflects a woman’s fertility potential. The expression of the AMH gene and AMH production increase until the follicle reaches a diameter of 8 mm. The role of estradiol in regulating AMH expression is considered significant, mediated through the estrogen receptor β present in the granulosa cells [26]. AMH levels can provide valuable information about a woman’s ovarian function and reproductive health. An optimal AMH level for IVF generally falls within the range of 7.0 to 28.5 pmol/L. Low AMH (below 7 pmol/L) indicates a lower ovarian reserve, which may suggest a poorer response to ovarian stimulation. The optimal range (7.0–28.5 pmol/L) for IVF suggests a good ovarian reserve, with a likely favorable response to stimulation and a reasonable number of eggs retrieved. High AMH (above 28.5 pmol/L) suggests a high ovarian reserve, but it can also indicate a risk of ovarian hyperstimulation syndrome, a potential complication of fertility treatment [27,28]. Therefore, assessing AMH levels before IVF is crucial for predicting ovarian response, optimizing treatment strategies, and improving the chances of successful pregnancy. By considering the role of AMH along with other influencing factors, clinicians can personalize IVF protocols, enhance treatment outcomes, and support individuals on their journey towards achieving parenthood. Our study aimed to investigate the influence of age, BMI, 25-OH vitamin D level, and thyroid autoimmunity on AMH in euthyroid non-PCOS women undergoing IVF.

## 2. Materials and Methods

This retrospective cross-sectional study was developed among female patients with regular menstrual cycles (21–35 days) who were investigated for couple infertility in Zygota Fertility Clinic Mures, including a five-year period (2018–2022).

### 2.1. Study Population

Our study population was carefully selected based on specific criteria, so the sample size was relatively small. Inclusion criteria were set for females aged 20 to 45 years, while exclusion criteria included abnormal TSH levels, elevated prolactin, PCOS, and systemic autoimmune disease or immunosuppressive treatment. These criteria were crucial in ensuring the relevance and accuracy of our findings. The study was conducted in accordance with the Declaration of Helsinki and approved by the Ethics Committee of the George Emil Palade University of Medicine, Pharmacy, Science and Technology of Targu Mures, number 3165/27 May 2024.

### 2.2. Studied Parameters

Study parameters included age, BMI, 25-OH vitamin D level, fT4, TSH, anti-thyroglobulin (ATG) or anti-thyroid peroxidase (ATPO) antibodies, FSH, luteinizing hormone (LH), estradiol, progesterone, total testosterone, and AMH concentrations measured in the early follicular phase (days 3–5). BMI was calculated by dividing the female’s weight in kilograms by her height in meters squared (kg/m^2^). The 25-OH vitamin D level was determined by a high-performance liquid chromatography technique (Bioclinica Inc., Timisoara, Romania) and considered if it was measured between May and September to exclude the winter decline in vitamin D. To define vitamin D status, 25-OH vitamin D levels between 20 and 100 ng/mL were considered adequate, and below 20 ng/mL was considered deficient. Serum TSH, fT4, ATPO-, anti-TGB-antibodies, LH, FSH, total testosterone, progesterone, and AMH determination were performed using the chemiluminescence/electrochemiluminescence immunoassay technique with specific CLIA/ECLIA kits in the same laboratory (Bioclinica Inc., Timisoara, Romania).

### 2.3. Statistical Analysis

The data were analyzed using GraphPad Prism 10.2.3 (GraphPad et al., San Diego, CA, USA) and IBM SPSS Statistics 27 (International Business Machines Corporation, Armonk, NY, USA). First, the Kolmogorov–Smirnov test was applied to evaluate the distribution type of the variables, ensuring the validity of our data. For normally distributed variables, data are eloquently presented as mean ± standard deviation (SD), while for non-normally distributed variables, we offer the medians with interquartile range (IQR). For comparing intergroup variables, we employed the *t*-test or the Mann–Whitney test as appropriate. We also employed the Spearman rank correlation coefficient (rho) to explore correlations between variables. A linear regression model for the AMH value outcome was performed in the presence of all studied parameters as covariables. We held ourselves to the high standard of a *p*-value < 0.05 to signify statistical significance.

## 3. Results

### 3.1. Descriptive Statistics for the Included Patients

A total of 52 female patients were enrolled in the study from the local fertility clinic. Their demographic, anthropometrical, and biochemical parameters are presented in Table 1. 

The patients were divided into subgroups depending on IVF outcome, their 25-OH vitamin D level, BMI, AMH level, and thyroid status. Autoimmune thyroiditis was considered present if anti-thyroid peroxidase and anti-thyroid antibody titer exceeded twice the upper limit of the normal range (34 microIU/mL in the case of ATPO and 11 IU/mL in the case of ATG), so the absolute antibody titer was not used for statistical analysis. The medical records of each patient were checked for specific ultrasonographic patterns of thyroiditis. A BMI between 18.5 and 24.9 kg/m^2^ was considered normal, between 25.0 and 29.9 kg/m^2^ overweight, while a BMI of 30.0 kg/m^2^ or higher was classified as obese. The results are shown in Table 2. There were 32 patients undergoing IVF, of which only 18 pregnancies were successful. As a result, pregnancy outcome factors were not studied in more depth due to the very small sample size.

### 3.2. Correlation Analysis between AMH and the Studied Parameters

The clinical and biochemical data were used for correlational analysis, and Spearman’s correlation coefficient (rho) was calculated for AMH and each studied parameter pair. The 95% confidence interval was added if the *p* value was below the significance level (0.05). The results are presented in Table 3. 

**Figure 1:**
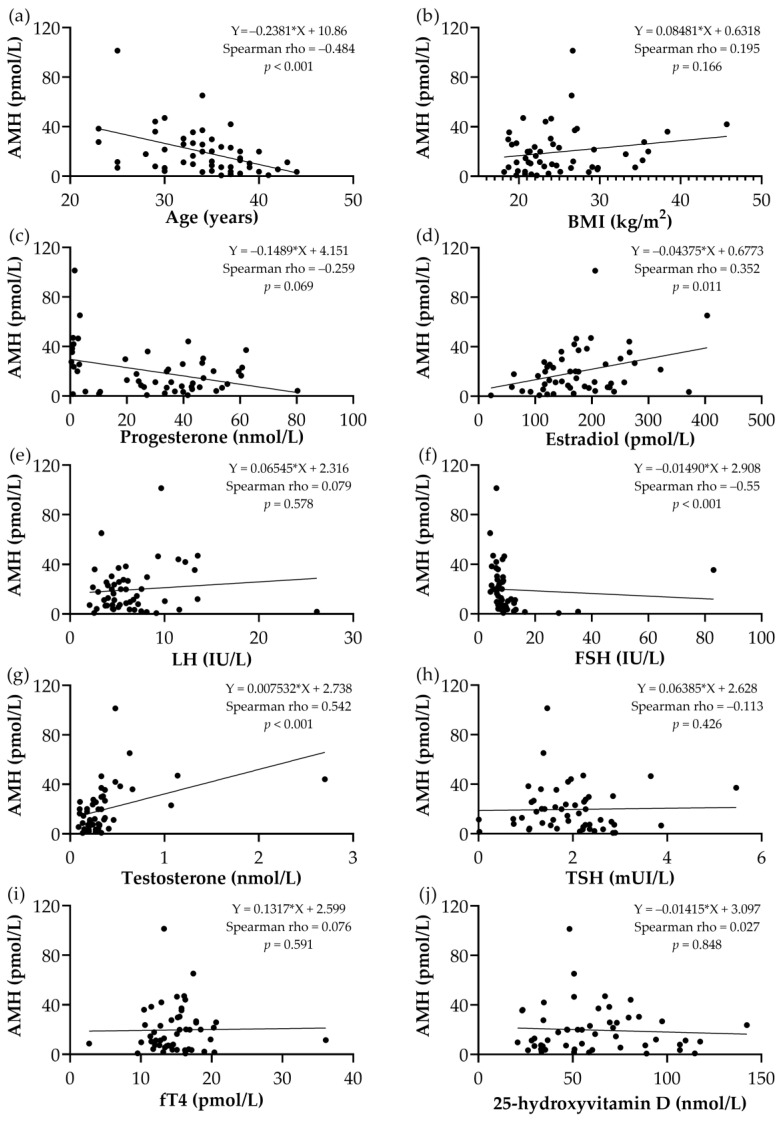
The relationship between clinical–biochemical parameters and AMH level. (**a**) Age and AMH correlation, (**b**) BMI and AMH correlation, (**c**) progesterone and AMH correlation, (**d**) estradiol and AMH correlation, (**e**) LH and AMH level correlation, (**f**) FSH and AMH level correlation, (**g**) testosterone and AMH level correlation, (**h**) TSH and AMH level correlation, (**i**) fT4 and AMH level correlation, and (**j**) AMH and 25-hydroxyvidamin D level correlation. BMI—body mass index; AMH—anti-Müllerian hormone; LH—luteinizing hormone; FSH—follicle-stimulating hormone; TSH—thyroid-stimulating hormone; fT4—free thyroxine; rho—Spearman’s correlation coefficient.

The scatter plot diagrams for AMH correlation with the studied parameters are presented in Figure 1.

### 3.3. Regression Model Analysis for AMH

The impact of many confounding factors required a study of their influence using a multivariable linear regression model. In this model, the AMH value serves as the outcome, while all other parameters studied act as covariates. The VIF (variance inflation factor) was under 3 for each variable, so there was no need to exclude either of them. The results of this regression analysis are shown in Table 4.

### 3.4. Comparative Analysis of the Studied Parameters Depending on the Presence of Autoimmune Thyroiditis

The studied population was separated into two categories according to the presence or absence of autoimmune thyroiditis. The hormonal panel and the clinical data were compared. The results are presented in Table 5.

## 4. Discussion

This study suggests that AMH levels show a negative correlation with age and a positive correlation with estradiol and testosterone levels in euthyroid non-PCOS females. There is, however, little influence of BMI, LH, FSH, and 25-OH vitamin D. 

AMH levels decline naturally with age as the number of antral follicles in the ovaries decreases. This decline starts from the mid-20 s and accelerates as a woman approaches her late 30 s and 40 s. By menopause, AMH levels are often undetectable, reflecting the exhaustion of the ovarian reserve. While there are variations based on ethnicity and geographical location, the highest median AMH level (4.42 ng/mL or 31.57 pmol/L) is seen in women under 25 years old, and the lowest (0.24 ng/mL or 1.7 pmol/L) is observed in women aged 50 years or older [10,29]. In addition to quantity, the quality of follicles also diminishes with age. This means that not only are fewer follicles available, but the remaining follicles are also less capable of producing viable eggs, contributing further to reduced AMH levels. In our study the patients’ mean age was 34.04 ± 4.95 years with a median value of AMH of 13.71 (IQR 20.61) pmol/L. Our data unequivocally shows that older patients consistently exhibit lower levels of AMH, while younger patients consistently demonstrate higher levels. We found a significant inverse correlation between age and AMH level, as shown in Figure 1a, which aligns with previous study results [10,11,30]. In our multiple linear regression analysis model, we also demonstrated a significant negative influence of age among the other covariables. Consistent with other studies [8,9,28], our results highlighted that age is a critical factor in determining a woman’s ovarian reserve, which refers to the quantity and quality of her remaining oocytes. 

The relationship between BMI and AMH has been explored in various studies, the majority of them suggesting an inverse relationship between BMI and AMH levels, especially in PCOS and central obesity patients [31,32]. A recent systematic review confirms that the combination of central obesity, high blood pressure, elevated triglycerides, elevated fasting glucose, and reduced HDL cholesterol has a negative impact on ovarian reserve [33]. Insulin resistance is strongly associated with obesity, type 2 diabetes, and metabolic syndrome, and serves as a key component of PCOS. Despite affecting fertility, PCOS is identified by elevated levels of AMH due to insulin actions in granulosa cells, which are marked by hyperandrogenism. Studies confirm higher AMH levels in PCOS than in non-PCOS women [31,34,35,36]. Our study excluded PCOS women, a decision made to avoid potential confounding factors in interpreting AMH levels in a small-sample-size study. Our findings did not show a significant correlation between BMI and AMH, a result consistent with other small-size studies [31,37]. A meta-analysis highlighted that the BMI’s impact on AMH levels is not conclusive; it depends on age, ethnicity, and other metabolic factors [38]. Similarly, no significant influence of BMI on ovarian reserve of non-PCOS women was found in our multiple linear regression model. This result underscores the need for further studies to clarify the BMI–AMH relationship, presenting an exciting opportunity for future research in this field, for example to study how the type of obesity (central or peripheral) influences AMH levels. 

The correlation between AMH and various reproductive hormones such as estradiol, progesterone, testosterone, LH, and FSH in the context of infertility is an exciting topic in the field of reproductive medicine. Lower levels of AMH often correspond with diminished ovarian reserve, associated with reduced estradiol production, which can hinder follicular development and ovulation, contributing to infertility. On the other hand, in conditions like PCOS, elevated AMH levels may be linked to high estradiol levels due to an increased number of antral follicles [39,40]. Studies suggest the importance of considering this correlation in conjunction with FSH and testosterone levels because androgens and growth factors influence the expression of AMH in the pre-antral stages in the granulosa cells, and testosterone is transformed via aromatase to estrogens [41,42,43,44]. Furthermore, as the follicle grows, this effect diminishes, and a shift to estradiol production occurs, which in turn stops AMH production from these follicles [45]. In our study, we found a significant positive correlation between estradiol and AMH, total testosterone and AMH, and a significant negative correlation between FSH and AMH levels. It is a well-known characteristic of ovarian physiology that estradiol and FSH correlate with AMH. However, some researchers consider that estrogens may not directly indicate ovarian reserve, but it does assess follicular growth [21,45]. Regarding the impact of total testosterone on AMH levels, it should be noted that 10 out of 52 patients had lower testosterone levels than normal, and none of them showed abnormally high levels. The significant positive correlation between total testosterone and AMH levels points towards an important role of testosterone in AMH production. A previous large study has also established a clear positive correlation between AMH and testosterone levels in infertile women without PCOS [43]. LH and progesterone level are rarely studied in correlation with AMH in female infertility, being hormonal factors implicated in ovulation and pregnancy outcome [46]. In our study there was no correlation found between AMH and LH levels. Furthermore, other researchers found no correlation between LH and AMH levels in healthy women [20]. The relationship between AMH and progesterone level, although not reaching the level of significance (*p* = 0.069), revealed that elevated progesterone lowered the AMH level. In our multivariate linear regression model, we found a significant positive impact of estrogens and a negative impact of the follicular phase progesterone on AMH levels, while total testosterone did not have any influence. Our findings suggest that estrogens, although not currently used as an ovarian reserve marker in infertility, could be a promising predictor of ovarian function in women without PCOS. This potential opens new opportunities for research in the field of reproductive endocrinology and infertility. We also found that elevated progesterone in the follicular phase has a negative impact on ovarian reserve; however, it is worth noting that other in vitro studies have reported the opposite effect [47]. It remains unclear how the elevated progesterone level affects the ovarium, but its effects on the endometrium might affect IVF success rates. Nonetheless, it was shown that progesterone elevation in the follicular phase has a detrimental impact on IVF outcome measures such as implantation, clinical pregnancy, and live birth rates [48].

Thyroid disorders are common in women undergoing in vitro fertilization, with the majority of studies reporting an inverse relation between TSH and AMH level [22,49,50]. Not only TSH level but the presence of thyroid autoantibodies can also alter ovarian reserve, disrupting IVF outcome [51,52,53,54]. To minimize the pathological TSH confounding effect on AMH, only euthyroid patients without levothyroxine treatment were included in our study. Neither in the correlation analysis nor in the multiple linear regression analysis were TSH and fT4 levels correlated with AMH, nor did they influence it significantly, although the presence of thyroid autoantibodies significantly influenced the TSH level. The median (IQR) value of TSH was significantly higher in the AIT positive group when compared to the AIT negative one. The conflicting result could be attributed to the deliberate exclusion of hypo- and hyperthyroid patients in the study design. Furthermore, it is important to acknowledge that autoimmunity was present in 63.5% of our euthyroid patients. It is well known that beginning from thirty years of age, the TSH starts to increase, but without the alteration of fT4. This is interpreted rather as a resetting of the hypophyseal–thyroid axis than a pathological state with need of therapeutical intervention. Obviously, this phenomenon is more pronounced in the elderly population [55]. If AMH declines and TSH rises with age, it could be a reasonable assumption that there is a theoretically negative correlation between these two parameters. However, the AMH decline is rapidly progressive in the premenopausal period, and the TSH increase becomes more evident only after menopause. A Chinese study from 2021 evaluated 496 female patients who were confronting infertility. The subjects with abnormal TSH value (3.63%) or thyroid autoimmunity (10.48%) were not excluded this time. The thyroid function panel was not significantly associated with AMH level [56]. 

The role of vitamin D in female infertility was extensively studied, and several reports indicate that adequate levels of 25-OH vitamin D are linked with increased progesterone synthesis [57] and improved conception and IVF success rate [58,59,60,61]. Although vitamin D stimulates AMH gene expression in vitro, there is no high-quality clinical evidence that serum 25-OH vitamin D levels are associated with serum AMH in any way. This study suggests that serum 25-OH vitamin D levels are not correlated with serum AMH levels. This is in agreement with a consistent number of previous studies where no correlation was found between these two parameters in any subgroup of infertile women [19,62,63]. Studies reporting a significant correlation between serum 25-OH vitamin D levels and serum AMH levels are statistically underpowered or refer to special subgroups of patients such as those over 45 years old [64]. On the other hand, the lack of correlation reported in this study does not contradict a potential beneficial effect of vitamin D supplementation on increasing AMH levels, conception rate, or IVF success rate. Due to the particular nature of both hormones, i.e., the autocrine/paracrine mode of action, serum concentrations might not reflect their function. Thus, future studies focusing on the association between AMH levels and vitamin D status should investigate the AMH levels in ovarian follicles in relation to different vitamin D status ranges [18,65]. Nevertheless, the evaluation of vitamin D status before IVF should be carried out because patients with a vitamin D deficit could benefit from supplementation.

The most important limitations of this study are its retrospective character, the small sample-size, and patients coming from a single IVF center, which may affect the generalizability of the findings to a larger population of infertile women, being applicable only to euthyroid and non-PCOS persons. While our study focused on primary variables in infertility, the absence of some key factors, such as antral ovarian follicle count, may have limited the depth of our analysis. Future studies should enlarge the number of patients and include other measurements to provide a more comprehensive understanding of AMH influencing factors.

## 5. Conclusions

This study concludes that multiple factors influence AMH levels, indicative of ovarian reserve. Age remains the most robust predictor, with a clear inverse relationship. AMH levels might also be affected by changing levels of sexual hormones, including estradiol, total testosterone, and FSH. On the other hand, body mass index and vitamin D status seem not to play a notable role in determining ovarian reserve in patients who are not suffering from polycystic ovary syndrome. Furthermore, thyroid health, both in terms of TSH levels and the presence of thyroiditis, does not significantly affect AMH when infertile patients are euthyroid. These findings collectively suggest that a holistic approach considering age, weight, hormonal balance, nutritional status, and thyroid health is essential for understanding and potentially managing ovarian reserve.

## Figures and Tables

**Table 1:** Description of the study sample demographic, anthropometric, and laboratory data.

Parameters (Unit of Measurement; Normal Range *)	Mean ± SD/Median (IQR)
Age (years)	34.04 ± 4.95
BMI (kg/m^2^)	23.48 (IQR 6.34)
AMH (pmol/L)	13.71 (IQR 20.61)
Progesterone (nmol/L; 1.59–63.60)	33.76 (IQR 38.55)
Estradiol (pmol/L; 69.75–513.99)	168.5 ± 75.05
LH (IU/L; 2.3–10.0)	5.51 (IQR 3.86)
FSH (IU/L; 1.1–9.5)	8.06 (IQR 2.77)
Total testosterone (nmol/L; 0.18–2.80)	0.28 (IQR 0.17)
TSH (mIU/L; 0.5–4.8)	1.93 ± 0.92
fT4 (pmol/L; 9.53–23.17)	14.84 (IQR 4.0)
25-OH vitamin D (nmol/L)	60.93 ± 28.49

N—total number of patients; SD—standard deviation; IQR—interquartile range; BMI—body mass index; AMH—anti-Müllerian hormone; LH—luteinizing hormone; FSH—follicle-stimulating hormone; TSH—thyroid-stimulating hormone; fT4—free thyroxine. * Laboratory normal range was included where applicable for diagnostic purposes.

**Table 2:** Clinical characteristics of the patients.

Studied Parameter	Outcome	Absolute and Relative Frequency (n/N, %)
IVF	No IVF performed, only infertility consultation	20/52 (38.5%)
	Unsuccessful IVF	14/52 (26.9%)
	Successful IVF	18/52 (34.6%)
Vitamin D status	Adequate vitamin D level (>50 nmol/L)	32/52 (61.5%)
	Deficient vitamin D (<50 nmol/L)	20/52 (38.5%)
Thyroid status	TSH < 2.5 mIU/L	42/52 (80.8%)
	TSH > 2.5 mIU/L	10/52 (19.2%)
Ovarian reserve	Adequate ovarian reserve (AMH > 7 pmol/L)	37/52 (71.2%)
	Low ovarian reserve (AMH < 7 pmol/L)	15/52 (28.8%)
Body weight	Normal weight	19/52 (36.5%)
	Overweight and obese	33/52 (63.5%)
Autoimmune thyroiditis	Present	33/52 (63.5%)
	Absent	19/52 (36.5%)

N—total number of patients (52); IVF—in vitro fertilization; AMH—anti-Müllerian hormone; LH—luteinizing hormone; FSH—follicle-stimulating hormone; TSH—thyroid-stimulating hormone; fT4—free thyroxine.

**Table 3:** Correlation between AMH level and the studied parameters.

		Age	BMI	Progesterone	Estradiol	LH	FSH	Total Testosterone (nmol/L)	TSH	fT4	25-OH Vitamin D
AMH	Rho	−0.484 (−0.673 to −0.236)	0.195	−0.254	0.352 (0.079 to 0.575)	0.079	−0.550 (−0.719 to −0.318)	0.542 (0.309 to 0.714)	−0.113	0.076	0.027
	*p* (2-tailed)	<0.001 *	0.166	0.069	0.011 *	0.578	<0.001 *	<0.001 *	0.426	0.591	0.848

Rho—Spearman’s correlation coefficient; BMI—body mass index; AMH—anti-Müllerian hormone; LH—luteinizing hormone; FSH—follicle-stimulating hormone; TSH—thyroid-stimulating hormone; fT4—free thyroxine; 25-OH vitamin D—25-hydroxivitamin D; * statistically significant.

**Table 4:** Linear regression model for AMH outcome in the presence of the studied covariables.

	Unstandardized Coefficients		Standardized Coefficient	t	*p*
	B	SE	β		
(Constant)	8.494	3.543		2.397	0.021 *
Age (years)	−0.243	0.066	−0.459	−3.678	0.001 *
BMI (kg/m^2^)	0.048	0.064	0.107	0.753	0.456
Progesterone (nmol/L)	−0.121	0.053	−0.303	−2.274	0.028 *
Estradiol (pmol/L)	0.052	0.016	0.403	3.307	0.002 *
LH (IU/L)	0.068	0.087	0.105	0.774	0.443
FSH (IU/L)	−0.031	0.032	−0.134	−0.960	0.343
Testosterone (nmol/L)	−0.027	0.031	−0.108	−0.886	0.381
TSH (mIU/L)	0.489	0.341	0.172	1.433	0.160
fT4 (pmol/L)	−0.834	0.945	−0.108	−0.882	0.383
25-hydroxivitamin D (nmol/L)	−0.002	0.032	−0.008	−0.055	0.957

B—unstandardized estimated coefficient; SE—standard error; β—regression coefficient; t—beta coefficient/standard error; BMI—body mass index; AMH—anti-Müllerian hormone; LH—luteinizing hormone; FSH—follicle-stimulating hormone; TSH—thyroid-stimulating hormone; fT4—free thyroxine; * statistically significant.

**Table 5:** Clinical and hormonal data comparison in AIT and non-AIT patients.

	AIT Present n = 33	AIT Absent n = 19	*p*	Test
Age (years) mean ± SD	34.67 ± 4.68	32.95 ± 5.33	0.231	Independent-samples *t*-test
BMI (kg/m^2^) median (IQR)	23.3 (5.51)	24.65 (13.33)	0.747	Mann–Whitney
AMH (pmol/L) median (IQR)	11.43 (21.64)	14.57 (18.93)	0.537	Mann–Whitney
Progesterone (nmol/L) mean ± SD	33.48 ± 19.27	23.64 ± 22.47	0.101	Independent-samples *t*-test
Estradiol (pmol/L) mean ± SD	182.8 ± 82.55	159.6 ± 59.01	0.287	Independent-samples *t*-test
LH (IU/L) median (IQR)	5.19 (3.43)	5.91 (5.79)	0.857	Mann–Whitney
FSH (IU/L) median (IQR)	8.03 (2.75)	8.11 (3.6)	0.676	Mann–Whitney
Total testosterone (nmol/L) median (IQR)	0.3 (0.15)	0.26 (0.2)	0.655	Mann–Whitney
TSH (mIU/L) median (IQR)	2.22 (1.01)	1.4 (0.75)	0.002 *	Mann–Whitney
fT4 (pmol/L) median (IQR)	15.22 (3.48)	13.29 (5.29)	0.121	Mann–Whitney
25-hydroxyvitamin D (nmol/L) mean ± SD	63.98 ± 29.64	55.63 ± 26.49	0.314	Independent-samples *t*-test

SD—standard deviation; IQR—interquartile range; BMI—body mass index; AMH—anti-Müllerian hormone; LH—luteinizing hormone; FSH—follicle-stimulating hormone; TSH—thyroid-stimulating hormone; fT4—free thyroxine; AIT—autoimmune thyroiditis; * statistically significant.

## Data Availability

The data presented in this study are available on request from the corresponding author.
